# Dynamic Analysis and Optimal Control of Rumor Spreading Model with Recurrence and Individual Behaviors in Heterogeneous Networks

**DOI:** 10.3390/e24040464

**Published:** 2022-03-27

**Authors:** Xinru Tong, Haijun Jiang, Xiangyong Chen, Shuzhen Yu, Jiarong Li

**Affiliations:** 1 College of Mathematics and System Science, Xinjiang University, Urumqi 830046, China; tongxinru11@163.com (X.T.); yusz0505@163.com (S.Y.); ljrmath@xju.edu.cn (J.L.); 2 School of Automation and Electrical Engineering, Linyi University, Linyi 276007, China; chenxiangyong@lyu.edu.cn

**Keywords:** rumor propagation, heterogeneous networks, rumor recurrence, individual behaviors, optimal control, model application

## Abstract

This paper is devoted to investigating the impact of the recurrence of rumors and individual behaviors and control strategies related to rumor spreading in online social networks. To do this, a novel susceptible-hesitating-infected-latent-recovered (SHILR) rumor propagation model in heterogeneous networks is presented. Firstly, based on the relevant mean-field equations of the model, the threshold value is examined to demonstrate the existence and stability of rumor-free/spreading equilibrium with the help of the algebraic method. Secondly, the global stabilities of the equilibria are analyzed through applying Lyapunov stability theory and LaSalle’s invariance principle. Next, the optimal control is proposed by taking advantage of Pontryagin’s maximum principle for reducing the number of infected individuals with minimum cost. Moreover, some numerical examples are carried out to test the theoretical results. Finally, combined with practice, a model application is presented.

## 1. Introduction

Rumors are usually interpreted as an unconfirmed statement of events or problems of public interest, which are spread through various channels [1,2]. With widespread internet access and use, people are exposed to a large number of online rumors. For example, on 31 January 2020, a rumor claiming “Shuanghuanglian” oral solution can suppress COVID-19 resulted in some domestic pharmacies to quickly sell out of “Shuanghuanglian”-related products, causing market chaos and bringing a series of negative effects [3]. Therefore, it is very important to explore the dynamics of rumor propagation in order to provide theoretical guidance for curbing rumors.

Since the dynamics of rumor spreading are highly similar to that of infectious epidemics, many scholars draw lessons from the compartmental modeling method in biological infectious diseases. Some scholars consider features of human social behavior as the basis for establishing rumor spreading models and extrapolate laws inherent to rumor spreading. The classic rumor spreading models can be traced back to the DK model [4] proposed by Daley and Kendall in 1965 and the MT model [5] proposed by Maki and Thomson. With these as their foundation, many scholars have studied the dynamics of rumor propagation and created many meaningful rumor propagation models. In particular, Zanettle first introduced complex networks into a rumor propagation model and established a model in small-world networks [6,7]. His research shows that the structure of networks has a dramatic effect on rumor spreading. It is widely known that complex networks are divided into homogeneous networks and heterogeneous networks according to whether the nodes of the network are of the same degree. In addition, a large number of rumor-spreading models have been discussed in homogeneous networks [8,9,10,11,12,13,14,15].

Actually, it is impossible for each individual to have contact with the same number of people. Therefore, propagation of rumors is too complex to simply assume that the number of people contacted by each individual is equal. In real life, everyone has a different number of friends. Those who have good social skills have a greater impact on the spreading of rumors. Thus, it is necessary to study the spread of rumors in heterogeneous networks. Recently, some research of rumor propagation in heterogeneous networks have been published [16,17,18,19,20]. In [17], Hosseini et al. introduced a new dynamic rumor propagation model of malware propagation in heterogeneous networks. Ma et al. proposed a novel rumor spreading model in heterogeneous networks by considering the individuals’ subjective judgment and diverse characteristics [19]. Zhu et al. considered network supervision and network topology, then proposed a novel susceptible-propagating-recovery (SIR) rumor propagation model with tome delay in both homogeneous and heterogeneous networks [20]. These works show that rumor propagation in heterogeneous networks is more interesting and complex. The rumor propagation model in heterogeneous networks could better reflect the real rumor propagation process.

In addition, it is of great practical significance to study the spreading mechanism of rumors in order to better understand and control rumor propagation. To describe the dynamics of rumor propagation more precisely, many scholars have considered various rumor spreading mechanisms, such as counterattack and self-resistance mechanism [21], trust mechanism [22], rational consciousness mechanism [23], latent mechanism [24], media reports mechanism [25], official refutation mechanism [26] and doubt mechanism [27]. It is worth noting that there is the possibility of recurrence in the process of rumor spreading. Yao et al. [28] considered the rumor spreading model with a rumor recurrence mechanism in homogeneous networks. Recurrence refers to the fact that some individuals may cease spreading the rumor at one point in time but resume spreading it after unforeseen circumstances reignite their interest. In this paper, recurrence mechanisms are considered in heterogeneous networks.

Moreover, individual behaviors need to be taken into consideration in modeling, as different people will have different reactions to the same rumor [29]. Actually, if people hear rumors, they usually immediately decide their course of action. That is to say, an ignorant individual generally chooses one of three different behaviors when he hears a rumor. The first option is to believe and spread the rumor and become a spreader; the second option is to hesitate—unsure of whether or not to spread the rumor; the third option is to not spread the rumor, directly becoming a recovered individual. Based on this, individual behaviors are factored into rumor propagation models in heterogeneous networks. Furthermore, in [30,31], the authors think that if the content is more important and ambiguous, the rumor will spread faster. Therefore, on the basis of these research results, a new parameter is introduced to quantify the attractiveness of rumors to people, denoted by *m*. It could be regarded as the unity of the significance and fuzziness of rumor content. The larger the *m*, the greater the interested in the rumor.

In view of the negative impact of rumor spread, it is urgent to restrain rumor spreading. Therefore, optimal control is born at the moment. Optimal control is a control to make target index of the given system reach the maximum (or minimum) under the given constraint conditions. Applying optimal control to rumor propagation models usually means that it can cut down the number of rumor infected individuals with minimum control cost. Zhu et al. [32] considered optimal immune control strategy in rumor propagation process and achieved good inhibition effect. Li et al. [33] considered the optimal education strategy and reduce the number of rumor infected individuals effectively. It is well known that excessive control intensity will lead to unnecessary waste of resources while insufficient control intensity could not curb the spreading of rumors. Thus, optimal control is particularly important to restrain rumor propagation. In this paper, an optimal media coverage control strategy is studied.

Motivated by the above analysis, this paper is devoted to investigating the impact of the recurrence of rumors, individual behaviors and the optimal media coverage control strategy for rumor propagation in heterogeneous networks. The main contributions and research work of this paper can be generalized as follows:

(1) Different from [28], not only is the recurrence mechanism considered, but also individual behaviors and the attractiveness factor are taken into account in our models, which makes the models more general and more practical.
(2) The optimal media coverage control strategy is proposed based on Pontryagin’s Maximum Principle in order to restrain the propagation of the rumor with the lowest cost.
(3) A specific heterogeneous network for studying rumor propagation, such as a BA scale-free network, is established. Moreover, a practical example is presented to verify the feasibility of the established model, which makes the proposed model more complex but more practical.

The outline of the rest of the paper is arranged as follows. In Section 2, a novel SHILR model is presented in heterogeneous networks. In Section 3, the global asymptotic stabilities of equilibria are proved. In Section 4, the optimal media coverage control strategy is put forward. In Section 5, several numerical examples are carried out. In Section 6, application of the model with a practical example is provided. Section 7 gives conclusions.

## 2. Model Formulation

**Figure 1:**
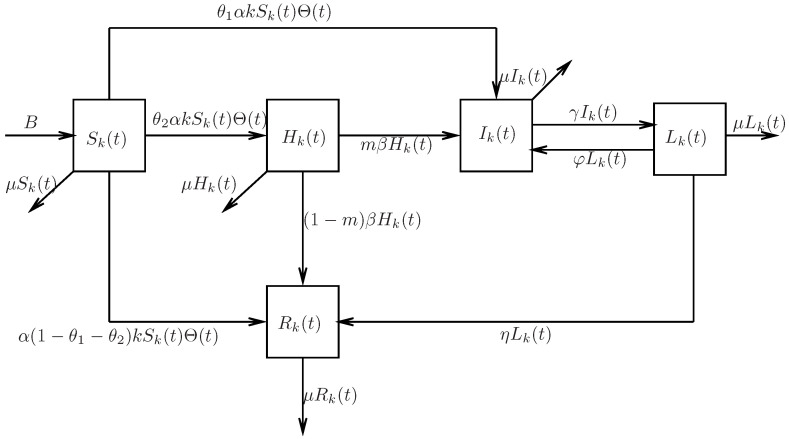
Structure of the SHILR rumor spreading process.

In this section, a SHILR rumor propagation model in heterogeneous networks is proposed. Considering the total population of N(t) and their contacts as complex networks, the concept of complex network is introduced, including nodes, edges and the degree [34] of nodes in networks. Based on complex network theory, rumor propagation in heterogeneous networks is discussed. Nodes represent individuals in social networks. The edges connecting two nodes in networks describe the contacts between individuals. The degree (k) of nodes in networks represents the number of the individuals’ own friends. Assume that the total population (N(t)) in networks is divided into five categories. Susceptible individuals (S(t)) mean the people who have not had contact with the rumor. Hesitating individuals (H(t)) represent the people who hear the rumor but hesitate to spread the rumor. Infected individuals (I(t)) denote the people who know and believe the rumor and spread it immediately. Latent individuals (L(t)) represent the people who cease spreading the rumor at the moment but might spread the rumor again. Recovered individuals (R(t)) denote the people who already know the rumor but do not spread it. Denote the density of susceptible, hesitating, infected, latent and recovered individuals with degree *k* at time *t* as $S_{k}\left(t\right)$, $H_{k}\left(t\right)$, $I_{k}\left(t\right)$, $L_{k}\left(t\right)$ and $R_{k}\left(t\right)$, respectively. In addition, it is assumed that the total population is variable over time in this paper. As shown in Figure 1, the rules of the SHILR rumor propagation model can be described as follows:

- Assume the whole group has a constant immigration rate *B*, and μ denotes the emigration rate.
- After a susceptible individual hears the rumor through contact with an infected individual with probability α, he has three possible choices. If he prefers to believe and spread the rumor, he will become an infected individual with probability $\theta_{1}$. If he is hesitant about spreading the rumor, he will become a hesitating individual with probability $\theta_{2}$. If he does not spread the rumor, he will develop into a recovered individual with probability $\left(1-\theta_{1}-\theta_{2}\right)$.
- After a period of time, the hesitating individual is infected with probability β. The hesitating individual becomes an infected individual with probability mβ (*m* represents the attractiveness of rumor), and he becomes a recovered individual with probability (1−m)β.
- As for infected individuals, they will turn into latent state when the rumor is found to be fabricated. Latent state is a particular state in which some individuals may totally lose their interest in rumor propagation and then become recovered individuals with probability η, but other individuals may regain their interest in rumor propagation as a result of unforeseen events, meaning they become infected individuals again with probability φ.

**Remark** **1.** *From Figure 1, it is noticeable that the rules of rumor propagation considered in this paper differ from the previous articles [8,9,10,11,12,13,14,15,16,17,18,19,20]. The different behaviors of susceptible individuals and the attractiveness of rumors are discussed.*

Thus, with the assistance of the heterogeneous mean-field method [35,36], the dynamics of the SHILR rumor propagation model in heterogeneous networks with rumor recurrence and individual behaviors is presented:

(1) $$\left\{\begin{array}{c} \frac{dS_{k}\left(t\right)}{dt}=B-\alpha kS_{k}\left(t\right)\Theta \left(t\right)-\mu S_{k}\left(t\right),\\ \frac{dH_{k}\left(t\right)}{dt}=\theta_{2}\alpha kS_{k}\left(t\right)\Theta \left(t\right)-\left(\beta +\mu \right)H_{k}\left(t\right),\\ \frac{dI_{k}\left(t\right)}{dt}=\theta_{1}\alpha kS_{k}\left(t\right)\Theta \left(t\right)+m\beta H_{k}\left(t\right)-\left(\gamma +\mu \right)I_{k}\left(t\right)+\varphi L_{k}\left(t\right),\\ \frac{dL_{k}\left(t\right)}{dt}=\gamma I_{k}\left(t\right)-\left(\varphi +\eta +\mu \right)L_{k}\left(t\right),\\ \frac{dR_{k}\left(t\right)}{dt}=\left(1-m\right)\beta H_{k}\left(t\right)+\left(1-\theta_{1}-\theta_{2}\right)\alpha kS_{k}\left(t\right)\Theta \left(t\right)+\eta L_{k}\left(t\right)-\mu R_{k}\left(t\right), \end{array}\right.$$

with the initial conditions

(2) $$\begin{array}{cc} \; & S_{k}\left(0\right)>0,\;\;H_{k}\left(0\right)>0,\;\;I_{k}\left(0\right)>0,\;\;L_{k}\left(0\right)>0,\;\;R_{k}\left(0\right)>0,\;\;k=1,2,\cdots ,n. \end{array}$$

Denote the total population density with degree *k* at time *t* as $N_{k}\left(t\right)$. Thus

$$N_{k}\left(t\right)=S_{k}\left(t\right)+H_{k}\left(t\right)+I_{k}\left(t\right)+L_{k}\left(t\right)+R_{k}\left(t\right).$$

Assume that B,μ,m>0 and $\alpha ,\beta ,\gamma ,\varphi ,\eta ,\theta_{1},\theta_{2},\left(1-\theta_{1}-\theta_{2}\right)\in \left(0,1\right)$. Θ(t) represents the probability that the neighbor of a node with degree *k* is infected at time *t*, which is described as

$$\Theta \left(t\right)=\sum_{i=1}^{n}p\left(i|k\right)I_{i}\left(t\right),\;\;i=1,2,\cdots ,n.$$

Here, p(i|k) is the conditional probability that a node with degree *k* is connected to a node with degree *i*. In this paper, degree-uncorrelated networks [37] are implemented, in which the conditional probability can be written as $p\left(i|k\right)=\frac{ip(i)}{\langle k\rangle }$, where $\langle k\rangle =\sum_{k=1}^{n}kp\left(k\right)$ is the average degree and p(i) is the degree distribution. Hence, substituting p(i|k) into Θ(t), one can obtain

(3) $$\begin{array}{c} \Theta \left(t\right)=\frac{1}{\langle k\rangle }\sum_{i=1}^{n}ip\left(i\right)I_{i}\left(t\right). \end{array}$$

**Remark** **2.** *In degree-uncorrelated networks, the existence of an edge between any two nodes is independent of the degree of these two nodes. That is to say, if the degree of the two endpoints of an edge randomly selected in a network is completely random, the network is a degree-uncorrelated network, otherwise it is a degree-correlated network. Thus, it can be seen that social networks are a degree-uncorrelated network. Actually, in real social networks, whether two person can become friends or not has nothing to do with the number of friends each of them has. Therefore, only degree-uncorrelated networks are considered in this paper.*

**Remark** **3.** *It is noticed that there is a big difference between heterogeneous networks and homogeneous networks. The degree of each node in homogeneous networks is the same, while it is different in heterogeneous networks. Actually, it is impossible for each person to have contact with the same number of individuals. In real life, everyone has a different number of friends. Those with good social skills have a greater impact on the spreading of rumors. Therefore, it is more reasonable to introduce heterogeneous networks to describe the rumor spreading process.*

**Remark** **4.** *In [38,39], rumor propagation models with a hesitation mechanism in heterogeneous networks were proposed. Compared with [38,39], in addition to a hesitation factor, individual behavior and a recurrence mechanism were also considered, which makes the model more general and more practical.*

## 3. The Basic Reproduction Number and the Analysis of Equilibria

In this section, the dynamics of SHILR rumor propagation in social networks will be analyzed. Because the first four equations are independent of the last one, one can consider the following system for simplification:

(4) $$\left\{\begin{array}{c} \frac{dS_{k}\left(t\right)}{dt}=B-\alpha kS_{k}\left(t\right)\Theta \left(t\right)-\mu S_{k}\left(t\right),\\ \frac{dH_{k}\left(t\right)}{dt}=\theta_{2}\alpha kS_{k}\left(t\right)\Theta \left(t\right)-\left(\beta +\mu \right)H_{k}\left(t\right),\\ \frac{dI_{k}\left(t\right)}{dt}=\theta_{1}\alpha kS_{k}\left(t\right)\Theta \left(t\right)+m\beta H_{k}\left(t\right)-\left(\gamma +\mu \right)I_{k}\left(t\right)+\varphi L_{k}\left(t\right),\\ \frac{dL_{k}\left(t\right)}{dt}=\gamma I_{k}\left(t\right)-\left(\varphi +\eta +\mu \right)L_{k}\left(t\right). \end{array}\right.$$

Obviously, system (4) has a rumor-free equilibrium:

$$E_{0}=\left\{\frac{B}{\mu },0,0,0,\cdots ,\frac{B}{\mu },0,0,0\right\}.$$

Furthermore, whether or not there is a rumor-spreading equilibrium in the system (4) will be discussed.

Define $R_{0}=\frac{\langle k^{2}\rangle }{\langle k\rangle }\frac{a_{3}\left(a_{1}\theta_{1}+m\beta \theta_{2}\right)\alpha B}{\mu a_{1}\left(a_{2}a_{3}-\varphi \gamma \right)}$, which is the basic reproduction number determined later.

**Lemma** **1.** When $R_{0}>1$, the system (4) has a unique rumor-spreading equilibrium $E^{*}=\left\{S_{1}^{*},H_{1}^{*},I_{1}^{*},L_{1}^{*},\cdots ,S_{n}^{*},H_{n}^{*},I_{n}^{*},L_{n}^{*}\right\}$.

**Proof** **of Lemma 1.**  Assume that $E^{*}=\left(S_{k}^{*},H_{k}^{*},I_{k}^{*},L_{k}^{*}\right)\left(k=1,2,\cdots ,n\right)$ is the rumor-spreading equilibrium, thus, the following equations hold:

(5) $$\begin{array}{c} \left\{\begin{array}{c} B-\alpha kS_{k}^{*}\Theta^{*}-\mu S_{k}^{*}=0,\\ \theta_{2}\alpha kS_{k}^{*}\Theta^{*}-\left(\beta +\mu \right)H_{k}^{*}=0,\\ \theta_{1}\alpha kS_{k}^{*}\Theta^{*}+m\beta H_{k}^{*}-\left(\gamma +\mu \right)I_{k}^{*}+\varphi L_{k}^{*}=0,\\ \gamma I_{k}^{*}-\left(\varphi +\eta +\mu \right)L_{k}^{*}=0. \end{array}\right. \end{array}$$

□

By solving (5), there exists:

(6) $$\begin{array}{cc} \; & S_{k}^{*}=\frac{B}{\alpha k\Theta^{*}+\mu },\;\;H_{k}^{*}=\frac{\theta_{2}\alpha kS_{k}^{*}\Theta^{*}}{\beta +\mu },\\ \; & I_{k}^{*}=\frac{a_{3}\left(a_{1}\theta_{1}+m\beta \theta_{2}\right)\alpha k\Theta^{*}}{a_{1}\left(a_{2}a_{3}-\varphi \gamma \right)}\frac{B}{\alpha k\Theta^{*}+\mu },\;\;L_{k}^{*}=\frac{\gamma I_{k}^{*}}{\varphi +\eta +\mu }, \end{array}$$

where $a_{1}=\beta +\mu ,a_{2}=\gamma +\mu ,a_{3}=\varphi +\eta +\mu .$ Based on (3), one can get the self-consistency equation as follows:

(7) $$\begin{array}{cc} \Theta^{*} & =\frac{1}{\langle k\rangle }\sum_{k=1}^{n}kp\left(k\right)I_{k}^{*}\\ \; & =\frac{1}{\langle k\rangle }\sum_{k=1}^{n}kp\left(k\right)\frac{a_{3}\left(a_{1}\theta_{1}+m\beta \theta_{2}\right)\alpha k\Theta^{*}}{a_{1}\left(a_{2}a_{3}-\varphi \gamma \right)}\frac{B}{\alpha k\Theta^{*}+\mu }. \end{array}$$

Now, construct the following auxiliary function:

$$\begin{array}{c} f\left(\Theta^{*}\right)=1-\frac{1}{\langle k\rangle }\sum_{k=1}^{n}kp\left(k\right)\frac{a_{3}\left(a_{1}\theta_{1}+m\beta \theta_{2}\right)\alpha k}{a_{1}\left(a_{2}a_{3}-\varphi \gamma \right)}\frac{B}{\alpha k\Theta^{*}+\mu }. \end{array}$$

It is clear that $\Theta^{*}f\left(\Theta^{*}\right)=0$ and there is a rumor-free equilibrium $E_{0}$ of system (4). In the following, the case of $f(\Theta^{*})=0$ with $\Theta^{*}\neq 0$ is discussed.

Clearly, based on the definition of $f(\Theta^{*})$, one can get:

$$\begin{array}{cc} \frac{df(\Theta^{*})}{d\Theta^{*}} & =\frac{1}{\langle k\rangle }\sum_{k=1}^{n}kp\left(k\right)\frac{a_{3}\left(a_{1}\theta_{1}+m\beta \theta_{2}\right)\alpha k}{a_{1}\left(a_{2}a_{3}-\varphi \gamma \right)}\frac{\alpha kB}{{\left(\alpha k\Theta^{*}+\mu \right)}^{2}}>0, \end{array}$$

$$\begin{array}{c} f\left(1\right)=1-\frac{1}{\langle k\rangle }\sum_{k=1}^{n}kp\left(k\right)\frac{a_{3}\left(a_{1}\theta_{1}+m\beta \theta_{2}\right)\alpha k}{a_{1}\left(a_{2}a_{3}-\varphi \gamma \right)}\frac{B}{\alpha k+\mu }. \end{array}$$

Since $0<\Theta^{*}<1,$ one can obtain:

$$\frac{a_{3}\left(a_{1}\theta_{1}+m\beta \theta_{2}\right)\alpha k\Theta^{*}}{a_{1}\left(a_{2}a_{3}-\varphi \gamma \right)}\frac{B}{\alpha k+\mu }<\frac{a_{3}\left(a_{1}\theta_{1}+m\beta \theta_{2}\right)\alpha k\Theta^{*}}{a_{1}\left(a_{2}a_{3}-\varphi \gamma \right)}\frac{B}{\alpha k\Theta^{*}+\mu }=I_{k}^{*}<1.$$

Therefore, $\frac{a_{3}\left(a_{1}\theta_{1}+m\beta \theta_{2}\right)\alpha k\Theta^{*}}{a_{1}\left(a_{2}a_{3}-\varphi \gamma \right)}\frac{B}{\alpha k+\mu }<1$ for all $\Theta^{*}\in \left(0,1\right).$

Let $g\left(\Theta^{*}\right)=\frac{a_{3}\left(a_{1}\theta_{1}+m\beta \theta_{2}\right)\alpha k\Theta^{*}}{a_{1}\left(a_{2}a_{3}-\varphi \gamma \right)}\frac{B}{\alpha k+\mu },$ based on continuity of $f(\Theta^{*})$, one can get:

$$\lim_{\Theta^{*}\to 1}g\left(\Theta^{*}\right)=\frac{a_{3}\left(a_{1}\theta_{1}+m\beta \theta_{2}\right)\alpha k}{a_{1}\left(a_{2}a_{3}-\varphi \gamma \right)}\frac{B}{\alpha k+\mu }<1.$$

Therefore, $f\left(1\right)=1-\frac{1}{\langle k\rangle }\sum_{k=1}^{n}kp\left(k\right)\frac{a_{3}\left(a_{1}\theta_{1}+m\beta \theta_{2}\right)\alpha k}{a_{1}\left(a_{2}a_{3}-\varphi \gamma \right)}\frac{B}{\alpha k+\mu }>0.$ Since $\frac{df(\Theta^{*})}{d\Theta^{*}}>0$ for all $\Theta^{*}$ and f(1)>0, the above indicates that $f(\Theta^{*})=0$ has a unique positive solution only when $\lim_{\Theta^{*}\to 0^{+}}f\left(\Theta^{*}\right)<0.$ Based on $\langle k^{2}\rangle =\sum_{k=1}^{n}k^{2}p\left(k\right)$, thus:

$$\begin{array}{cc} f(0) & =1-\frac{1}{\langle k\rangle }\sum_{k=1}^{n}kp\left(k\right)\frac{a_{3}\left(a_{1}\theta_{1}+m\beta \theta_{2}\right)\alpha k}{a_{1}\left(a_{2}a_{3}-\varphi \gamma \right)}\frac{B}{\mu }\\ \; & =1-\frac{1}{\langle k\rangle }\sum_{k=1}^{n}k^{2}p\left(k\right)\frac{a_{3}\left(a_{1}\theta_{1}+m\beta \theta_{2}\right)\alpha }{a_{1}\left(a_{2}a_{3}-\varphi \gamma \right)}\frac{B}{\mu }\\ \; & =1-\frac{\langle k^{2}\rangle }{\langle k\rangle }\frac{a_{3}\left(a_{1}\theta_{1}+m\beta \theta_{2}\right)\alpha B}{\mu a_{1}\left(a_{2}a_{3}-\varphi \gamma \right)}, \end{array}$$

that is

$$\frac{\langle k^{2}\rangle }{\langle k\rangle }\frac{a_{3}\left(a_{1}\theta_{1}+m\beta \theta_{2}\right)\alpha B}{\mu a_{1}\left(a_{2}a_{3}-\varphi \gamma \right)}>1.$$

Therefore, the basic reproduction number is defined as follows:

(8) $$\begin{array}{c} R_{0}=\frac{\langle k^{2}\rangle }{\langle k\rangle }\frac{a_{3}\left(a_{1}\theta_{1}+m\beta \theta_{2}\right)\alpha B}{\mu a_{1}\left(a_{2}a_{3}-\varphi \gamma \right)}. \end{array}$$

Thus, a unique positive solution exists if and only if $R_{0}>1$. In other words, when $R_{0}>1$, only one positive equilibrium $E^{*}=\left\{S_{1}^{*},H_{1}^{*},I_{1}^{*},L_{1}^{*},\cdots ,S_{n}^{*},H_{n}^{*},I_{n}^{*},L_{n}^{*}\right\}$ of system (4) exists. The proof is completed.

**Remark** **5.** *In this paper, the existence and uniqueness of positive solutions of the model are investigated to obtain the expression of basic reproduction number $R_{0}$. It is known that $R_{0}$ is a threshold condition to measure whether or not rumors spread. In other words, when $R_{0}>1$, there will be a rumor-spreading equilibrium. Therefore, one can deduce the basic reproductive number $R_{0}$ of the model by exploring the existence and uniqueness conditions of the rumor-spreading equilibrium. The expression of basic reproduction number $R_{0}$ obtained by this method is the same as the one by the next generation matrix [40].*

Based on (3), system (4) can be written as the following equivalent systems:

(9) $$\begin{array}{c} \left\{\begin{array}{c} \frac{dS_{k}\left(t\right)}{dt}=B-\alpha kS_{k}\left(t\right)\frac{1}{\langle k\rangle }\sum_{k=1}^{n}kp\left(k\right)I_{k}\left(t\right)-\mu S_{k}\left(t\right),\\ \frac{dH_{k}\left(t\right)}{dt}=\theta_{2}\alpha kS_{k}\left(t\right)\frac{1}{\langle k\rangle }\sum_{k=1}^{n}kp\left(k\right)I_{k}\left(t\right)-\left(\beta +\mu \right)H_{k}\left(t\right),\\ \frac{dI_{k}\left(t\right)}{dt}=\theta_{1}\alpha kS_{k}\left(t\right)\frac{1}{\langle k\rangle }\sum_{k=1}^{n}kp\left(k\right)I_{k}\left(t\right)+m\beta H_{k}\left(t\right)-\left(\gamma +\mu \right)I_{k}\left(t\right)+\varphi L_{k}\left(t\right),\\ \frac{dL_{k}\left(t\right)}{dt}=\gamma I_{k}\left(t\right)-\left(\varphi +\eta +\mu \right)L_{k}\left(t\right). \end{array}\right. \end{array}$$

**Theorem** **1.** *For system (9), the rumor-free equilibrium $E_{0}=\left\{\frac{B}{\mu },0,0,0,\cdots ,\frac{B}{\mu },0,0,0\right\}$ is globally asymptotically stable if $R_{0}<1$.*

**Proof** **of Theorem 1.**  Construct the Lyapunov function $W_{1}\left(t\right)$ as:

(10) $$\begin{array}{c} W_{1}\left(t\right)=\sum_{k=1}^{n}kp\left(k\right)\Upsilon_{k}\left(t\right), \end{array}$$

where $\Upsilon_{k}\left(t\right)=m\beta H_{k}\left(t\right)+a_{1}I_{k}\left(t\right)+\frac{a_{1}\varphi }{a_{3}}L_{k}\left(t\right).$

$$\begin{array}{cc} \frac{d\Upsilon_{k}\left(t\right)}{dt}= & m\beta \frac{dH_{k}\left(t\right)}{dt}+a_{1}\frac{dI_{k}\left(t\right)}{dt}+\frac{a_{1}\varphi }{a_{3}}\frac{dL_{k}\left(t\right)}{dt}\\ = & m\beta \left(\theta_{2}\alpha kS_{k}\left(t\right)\frac{1}{\langle k\rangle }\sum_{k=1}^{n}kp\left(k\right)I_{k}\left(t\right)-a_{1}H_{k}\left(t\right)\right)+\frac{a_{1}\varphi }{a_{3}}\left(\gamma I_{k}\left(t\right)-a_{3}L_{k}\left(t\right)\right)\\ \; & +a_{1}\left(\theta_{1}\alpha kS_{k}\left(t\right)\frac{1}{\langle k\rangle }\sum_{k=1}^{n}kp\left(k\right)I_{k}\left(t\right)+m\beta H_{k}\left(t\right)-a_{2}I_{k}\left(t\right)+\varphi L_{k}\left(t\right)\right)\\ = & m\beta \theta_{2}\alpha S_{k}\left(t\right)I_{k}\left(t\right)\frac{\langle k^{2}\rangle }{\langle k\rangle }-a_{1}m\beta H_{k}\left(t\right)+a_{1}\theta_{1}\alpha S_{k}\left(t\right)I_{k}\left(t\right)\frac{\langle k^{2}\rangle }{\langle k\rangle }\\ \; & +a_{1}m\beta H_{k}\left(t\right)-a_{1}a_{2}I_{k}\left(t\right)+a_{1}\varphi L_{k}\left(t\right)+\frac{a_{1}\varphi \gamma }{a_{3}}I_{k}\left(t\right)-a_{1}\varphi L_{k}\left(t\right)\\ = & m\beta \theta_{2}\alpha S_{k}\left(t\right)I_{k}\left(t\right)\frac{\langle k^{2}\rangle }{\langle k\rangle }+a_{1}\theta_{1}\alpha S_{k}\left(t\right)I_{k}\left(t\right)\frac{\langle k^{2}\rangle }{\langle k\rangle }-a_{1}a_{2}I_{k}\left(t\right)+\frac{a_{1}\varphi \gamma }{a_{3}}I_{k}\left(t\right)\\ = & \left[\frac{a_{3}\left(m\beta \theta_{2}+\theta_{1}a_{1}\right)\alpha S_{k}\left(t\right)\frac{\langle k^{2}\rangle }{\langle k\rangle }-a_{1}\left(a_{2}a_{3}-\varphi \gamma \right)}{a_{3}}\right]I_{k}\left(t\right)\\ \leq & \left[\frac{a_{3}\left(m\beta \theta_{2}+\theta_{1}a_{1}\right)\alpha B\frac{\langle k^{2}\rangle }{\langle k\rangle }}{\mu a_{3}}-\frac{a_{1}\left(a_{2}a_{3}-\varphi \gamma \right)}{a_{3}}\right]I_{k}\left(t\right)\\ = & \left(R_{0}-1\right)\frac{a_{1}\left(a_{2}a_{3}-\varphi \gamma \right)}{a_{3}}I_{k}\left(t\right). \end{array}$$

It is equivalent to the following:

(11) $$\begin{array}{c} \frac{dW_{1}\left(t\right)}{dt}=\sum_{k=1}^{n}kp\left(k\right)\left(R_{0}-1\right)\frac{a_{1}\left(a_{2}a_{3}-\varphi \gamma \right)}{a_{3}}I_{k}\left(t\right). \end{array}$$

As mentioned above in (11), $\frac{dW_{1}\left(t\right)}{dt}\leq 0$ when $R_{0}<1$. $\frac{dW_{1}\left(t\right)}{dt}=0$ equivalently implies that $S_{k}\left(t\right)=\frac{B}{\mu },H_{k}\left(t\right)=0,I_{k}\left(t\right)=0,L_{k}\left(t\right)=0$. Thus, in view of LaSalle’s Invariance Principle [41], one can get $\lim_{t\to \infty }S_{k}\left(t\right)=\frac{B}{\mu },\lim_{t\to \infty }H_{k}\left(t\right)=0,\lim_{t\to \infty }I_{k}\left(t\right)=0,\lim_{t\to \infty }L_{k}\left(t\right)=0.$ Thus, $E_{0}$ of system (9) is globally asymptotically stable when $R_{0}<1$. □

**Theorem** **2.** The rumor-spreading equilibrium $E^{*}=\left\{S_{1}^{*},H_{1}^{*},I_{1}^{*},L_{1}^{*},\cdots ,S_{n}^{*},H_{n}^{*},I_{n}^{*},L_{n}^{*}\right\}$ of system (9) is globally asymptotically stable if $R_{0}>1$ for the system (9).

**Proof** **of** **Theorem** **2.** Construct the Lyapunov function $W_{2}\left(t\right)$ as:

$$\begin{array}{c} W_{2}\left(t\right)=\sum_{k=1}^{n}kp\left(k\right)V_{k}\left(t\right). \end{array}$$

Denote

$$\begin{array}{cc} V_{k}\left(t\right)= & \left(m\beta \theta_{2}+a_{1}\theta_{1}\right)S_{k}^{*}g\left(\frac{S_{k}\left(t\right)}{S_{k}^{*}}\right)+m\beta H_{k}^{*}g\left(\frac{H_{k}\left(t\right)}{H_{k}^{*}}\right)+a_{1}I_{k}^{*}g\left(\frac{I_{k}\left(t\right)}{I_{k}^{*}}\right)\\ \; & +\frac{a_{1}\varphi }{a_{3}}L_{k}^{*}g\left(\frac{L_{k}\left(t\right)}{L_{k}^{*}}\right), \end{array}$$

where g(n)=n−1−lnn≥g(1)=0, for any n>0. Let

$$x=\frac{S_{k}\left(t\right)}{S_{k}^{*}},y=\frac{H_{k}\left(t\right)}{H_{k}^{*}},z=\frac{I_{k}\left(t\right)}{I_{k}^{*}},u=\frac{L_{k}\left(t\right)}{L_{k}^{*}}.$$

Then, substituting the rumor-spreading equilibrium into (9), one can obtain:

(12) $$\begin{array}{c} \left\{\begin{array}{c} B-\alpha kS_{k}^{*}\frac{1}{\langle k\rangle }\sum_{k=1}^{n}kp\left(k\right)I_{k}^{*}-\mu S_{k}^{*}=0,\\ \theta_{2}\alpha kS_{k}^{*}\frac{1}{\langle k\rangle }\sum_{k=1}^{n}kp\left(k\right)I_{k}^{*}-\left(\beta +\mu \right)H_{k}^{*}=0,\\ \theta_{1}\alpha kS_{k}^{*}\frac{1}{\langle k\rangle }\sum_{k=1}^{n}kp\left(k\right)I_{k}^{*}+m\beta H_{k}^{*}-\left(\gamma +\mu \right)I_{k}^{*}+\varphi L_{k}^{*}=0,\\ \gamma I_{k}^{*}-\left(\varphi +\eta +\mu \right)L_{k}^{*}=0. \end{array}\right. \end{array}$$

According to (9) and (12), one can get:

$$\begin{array}{cc} \frac{dV_{k}\left(t\right)}{dt}= & \left(m\beta \theta_{2}+a_{1}\theta_{1}\right)\left(1-\frac{S_{k}^{*}}{S_{k}\left(t\right)}\right)\frac{dS_{k}\left(t\right)}{dt}+m\beta \left(1-\frac{H_{k}^{*}}{H_{k}\left(t\right)}\right)\frac{dH_{k}\left(t\right)}{dt}\\ \; & +a_{1}\left(1-\frac{I_{k}^{*}}{I_{k}\left(t\right)}\right)\frac{dI_{k}\left(t\right)}{dt}+\frac{a_{1}\varphi }{a_{3}}\left(1-\frac{L_{k}^{*}}{L_{k}\left(t\right)}\right)\frac{dL_{k}\left(t\right)}{dt}\\ = & \left(m\beta \theta_{2}+a_{1}\theta_{1}\right)\left(1-\frac{S_{k}^{*}}{S_{k}\left(t\right)}\right)\left(B-\alpha kS_{k}\left(t\right)\frac{1}{\langle k\rangle }\sum_{k=1}^{n}kp\left(k\right)I_{k}\left(t\right)-\mu S_{k}\left(t\right)\right)\\ \; & +m\beta \left(1-\frac{H_{k}^{*}}{H_{k}\left(t\right)}\right)\left(\theta_{2}\alpha kS_{k}\left(t\right)\frac{1}{\langle k\rangle }\sum_{k=1}^{n}kp\left(k\right)I_{k}\left(t\right)-\left(\beta +\mu \right)H_{k}\left(t\right)\right)\\ \; & +a_{1}\left(1-\frac{I_{k}^{*}}{I_{k}\left(t\right)}\right)(\theta_{1}\alpha kS_{k}\left(t\right)\frac{1}{\langle k\rangle }\sum_{k=1}^{n}kp\left(k\right)I_{k}\left(t\right)+m\beta H\left(t\right)-\left(\gamma +\mu \right)I_{k}\left(t\right)\\ \; & +\varphi L_{k}\left(t\right))+\frac{a_{1}\varphi }{a_{3}}\left(1-\frac{L_{k}^{*}}{L_{k}\left(t\right)}\right)\left(\gamma I_{k}\left(t\right)-\left(\varphi +\eta +\mu \right)L_{k}\left(t\right)\right)\\ = & \left(m\beta \theta_{2}+a_{1}\theta_{1}\right)\left(1-\frac{S_{k}^{*}}{S_{k}\left(t\right)}\right)(\alpha kS_{k}^{*}\left(t\right)\frac{1}{\langle k\rangle }\sum_{k=1}^{n}kp\left(k\right)I_{k}^{*}\left(t\right)+\mu S_{k}^{*}\left(t\right)-\alpha kS_{k}\left(t\right)\\ \; & \times \frac{1}{\langle k\rangle }\sum_{k=1}^{n}kp\left(k\right)I_{k}\left(t\right)-\mu S_{k}\left(t\right))+m\beta \left(1-\frac{H_{k}^{*}}{H_{k}\left(t\right)}\right)(\theta_{2}\alpha kS_{k}\left(t\right)\frac{1}{\langle k\rangle }\sum_{k=1}^{n}kp\left(k\right)\\ \; & \times I_{k}\left(t\right)-\frac{\theta_{2}\alpha kS_{k}^{*}\frac{1}{\langle k\rangle }\sum_{k=1}^{n}kp\left(k\right)I_{k}^{*}}{H_{k}^{*}}H_{k}\left(t\right))+a_{1}\left(1-\frac{I_{k}^{*}}{I_{k}\left(t\right)}\right)(\theta_{1}\alpha kS_{k}\left(t\right)\frac{1}{\langle k\rangle }\\ \; & \times \sum_{k=1}^{n}kp\left(k\right)I_{k}\left(t\right)+m\beta H_{k}\left(t\right)+\varphi L_{k}\left(t\right)\\ \; & -\frac{\theta_{1}\alpha kS_{k}^{*}\frac{1}{\langle k\rangle }\sum_{k=1}^{n}kp\left(k\right)I_{k}^{*}+m\beta H_{k}^{*}+\varphi L_{k}^{*}}{I_{k}^{*}}I_{k}\left(t\right))\\ \; & +\frac{a_{1}\varphi }{a_{3}}\left(1-\frac{L_{k}^{*}}{L_{k}\left(t\right)}\right)\left(\frac{a_{3}L_{k}^{*}}{I_{k}^{*}}I_{k}\left(t\right)-a_{3}L_{k}\left(t\right)\right)\\ = & \left(m\beta \theta_{2}+a_{1}\theta_{1}\right)\left(1-\frac{1}{x}\right)\alpha kS_{k}^{*}\frac{1}{\langle k\rangle }\sum_{k=1}^{n}kp\left(k\right)I_{k}^{*}\left(1-xz\right)\\ \; & +\left(m\beta \theta_{2}+a_{1}\theta_{1}\right)\left(1-\frac{1}{x}\right)\mu S_{k}^{*}\left(1-x\right)+m\beta \left(1-\frac{1}{y}\right)\theta_{2}\alpha kS_{k}^{*}\frac{1}{\langle k\rangle }\sum_{k=1}^{n}kp\left(k\right)\\ \; & \times I_{k}^{*}\left(xz-y\right)+a_{1}\left(1-\frac{1}{z}\right)\theta_{1}\alpha kS_{k}^{*}\frac{1}{\langle k\rangle }\sum_{k=1}^{n}kp\left(k\right)I_{k}^{*}\left(xz-z\right)+a_{1}\left(1-\frac{1}{z}\right)\\ \; & \times m\beta H_{k}^{*}\left(y-z\right)+a_{1}\left(1-\frac{1}{z}\right)\varphi L_{k}^{*}\left(u-z\right)+\frac{a_{1}\varphi }{a_{3}}\left(1-\frac{1}{u}\right)a_{3}L_{k}^{*}\left(z-u\right)\\ = & \left(m\beta \theta_{2}+a_{1}\theta_{1}\right)\left(1-\frac{1}{x}-xz+z\right)\alpha kS_{k}^{*}\frac{1}{\langle k\rangle }\sum_{k=1}^{n}kp\left(k\right)I_{k}^{*}\\ \; & +\left(m\beta \theta_{2}+a_{1}\theta_{1}\right)\left(1-\frac{1}{x}-x+1\right)\mu S_{k}^{*}+m\beta \theta_{2}\alpha kS_{k}^{*}\frac{1}{\langle k\rangle }\sum_{k=1}^{n}kp\left(k\right)I_{k}^{*}\\ \; & \times \left(1-\frac{xz}{y}+xz-y\right)+a_{1}\theta_{1}\alpha kS_{k}^{*}\frac{1}{\langle k\rangle }\sum_{k=1}^{n}kp\left(k\right)I_{k}^{*}\left(xz-z-x+1\right)\\ \; & +a_{1}m\beta H_{k}^{*}\left(y-z-\frac{y}{z}+1\right)+a_{1}\varphi L_{k}^{*}\left(u-z-\frac{u}{z}+1\right)\\ \; & +a_{1}\varphi L_{k}^{*}\left(z-u-\frac{z}{u}+1\right)\\ = & 2\left(m\beta \theta_{2}+a_{1}\theta_{1}\right)\alpha kS_{k}^{*}\frac{1}{\langle k\rangle }\sum_{k=1}^{n}kp\left(k\right)I_{k}^{*}+2\left(m\beta \theta_{2}+a_{1}\theta_{1}\right)\mu S_{k}^{*}+a_{1}m\beta H_{k}^{*}\\ \; & +2a_{1}\varphi L_{k}^{*}-\left[\mu S_{k}^{*}\left(m\beta \theta_{2}+a_{1}\theta_{1}\right)+a_{1}\theta_{1}\alpha kS_{k}^{*}\frac{1}{\langle k\rangle }\sum_{k=1}^{n}kp\left(k\right)I_{k}^{*}\right]x\\ \; & -\left[\mu S_{k}^{*}\left(m\beta \theta_{2}+a_{1}\theta_{1}\right)+\left(a_{1}\theta_{1}+m\beta \theta_{2}\right)\alpha kS_{k}^{*}\frac{1}{\langle k\rangle }\sum_{k=1}^{n}kp\left(k\right)I_{k}^{*}\right]\frac{1}{x}\\ \; & +\left[a_{1}m\beta H_{k}^{*}-m\beta \theta_{2}\alpha kS_{k}^{*}\frac{1}{\langle k\rangle }\sum_{k=1}^{n}kp\left(k\right)I_{k}^{*}\right]y+[m\beta \theta_{2}\alpha kS_{k}^{*}\frac{1}{\langle k\rangle }\sum_{k=1}^{n}kp\left(k\right)\\ \; & \times I_{k}^{*}-a_{1}m\beta H_{k}^{*}]z-m\beta \theta_{2}\alpha kS_{k}^{*}\frac{1}{\langle k\rangle }\sum_{k=1}^{n}kp\left(k\right)I_{k}^{*}\frac{xz}{y}-a_{1}m\beta H_{k}^{*}\frac{y}{z}-a_{1}\varphi L_{k}^{*}\frac{u}{z}\\ \; & -a_{1}\varphi L_{k}^{*}\frac{z}{u}\\ = & m\beta \theta_{2}\alpha kkS_{k}^{*}\frac{1}{\langle k\rangle }\sum_{k=1}^{n}kp\left(k\right)I_{k}^{*}\left(2-\frac{1}{x}-y+z-\frac{xz}{y}\right)+a_{1}\theta_{1}\alpha kS_{k}^{*}\frac{1}{\langle k\rangle }\sum_{k=1}^{n}kp\left(k\right)\\ \; & \times I_{k}^{*}\left(2-x-\frac{1}{x}\right)+\mu S_{k}^{*}\left(m\beta \theta_{2}+a_{1}\theta_{1}\right)\left(2-x-\frac{1}{x}\right)+a_{1}m\beta H_{k}^{*}\left(1+y-z-\frac{y}{z}\right)\\ \; & +a_{1}\varphi L_{k}^{*}\left(2-\frac{u}{z}-\frac{z}{u}\right). \end{array}$$

According to the second equation of (12), one can know:

$$m\beta \theta_{2}\alpha kS_{k}^{*}\frac{1}{\langle k\rangle }\sum_{k=1}^{n}kp\left(k\right)I_{k}^{*}=a_{1}m\beta H_{k}^{*},\;\;i.e.,\;\;m\beta \theta_{2}\alpha \frac{\langle k^{2}\rangle }{\langle k\rangle }S_{k}^{*}I_{k}^{*}=a_{1}m\beta H_{k}^{*},$$

then

$$\begin{array}{cc} \frac{dV_{k}\left(t\right)}{dt}= & m\beta \theta_{2}\alpha \frac{\langle k^{2}\rangle }{\langle k\rangle }S_{k}^{*}I_{k}^{*}\left(3-\frac{1}{x}-\frac{xz}{y}-\frac{y}{z}\right)+a_{1}\theta_{1}\alpha \frac{\langle k^{2}\rangle }{\langle k\rangle }S_{k}^{*}I_{k}^{*}\left(2-x-\frac{1}{x}\right)\\ \; & +\mu S_{k}^{*}\left(m\beta \theta_{2}+a_{1}\theta_{1}\right)\left(2-x-\frac{1}{x}\right)+a_{1}\varphi L_{k}^{*}\left(2-\frac{u}{z}-\frac{z}{u}\right). \end{array}$$

It is equivalent to:

(13) $$\begin{array}{cc} \frac{dW_{2}\left(t\right)}{dt}= & \sum_{k=1}^{n}kp\left(k\right)[m\beta \theta_{2}\alpha \frac{\langle k^{2}\rangle }{\langle k\rangle }S_{k}^{*}I_{k}^{*}\left(3-\frac{1}{x}-\frac{xz}{y}-\frac{y}{z}\right)+a_{1}\theta_{1}\alpha \frac{\langle k^{2}\rangle }{\langle k\rangle }S_{k}^{*}I_{k}^{*}\left(2-x-\frac{1}{x}\right)\\ \; & +\mu S_{k}^{*}\left(m\beta \theta_{2}+a_{1}\theta_{1}\right)\left(2-x-\frac{1}{x}\right)+a_{1}\varphi L_{k}^{*}\left(2-\frac{u}{z}-\frac{z}{u}\right)]. \end{array}$$

It is worth noting that the inequality between an arithmetic mean and a geometric mean is as follows:

$$\frac{x_{1}+x_{2}+\cdots +x_{n}}{n}\geq \sqrt[{n}]{x_{1}x_{2}\cdots x_{n}}.$$

As mentioned above in (13), $\frac{dW_{2}\left(t\right)}{dt}\leq 0$. $\frac{dW_{2}\left(t\right)}{dt}=0$ equivalently implies that $S_{k}\left(t\right)=S_{k}^{*},H_{k}\left(t\right)=H_{k}^{*},I_{k}\left(t\right)=I_{k}^{*},L_{k}\left(t\right)=L_{k}^{*}$. Thus, in view of LaSalle’s Invariance Principle, $E^{*}$ is globally asymptotically stable when $R_{0}>1$. □

**Remark** **6.** *Compared to [28], besides the heterogeneous network under consideration, a stricter proof about the global asymptotic stabilities of rumor-free and rumor-spreading equilibria are given by constructing the Lyapunov function and using LaSalle’s Invariance Principle in this paper.*

## 4. Optimal Control

In this section, an optimal control problem for system (9) is proposed in order to reduce the number of infected individuals with minimum cost. The control strategy mentioned in this paper is media coverage. It is widely known that rumor propagation often stems from the lack of circulation of true information. When there is a lack of authoritative information, rumors are easy to spread widely. Therefore, official media coverage adopted by government departments is particularly important. The media can conduct in-depth reports and sort out the truth of the matter through various channels such as television, newspapers and the internet, so that the public can recognize and identify false news. In this way, the effect of controlling the spread of rumors is ultimately achieved. Let $U_{k}\left(t\right)$ represent the strength to control rumors through the use of media coverage. This measure can decrease the number of latent individuals who become infected individuals, reducing the probability that rumors recur. With the help of Pontryagin’s Maximum Principle [42], a Lebesgue-square integrable control function $U_{k}\left(t\right)\in \Delta$ is introduced, where $\Delta =\{U_{k}\left(t\right)|0\leq U_{k}\left(t\right)\leq 1,t\in \left[0,T\right],k=1,2,\cdots ,n\}$. Δ denotes the set of feasible controls and *T* is the ending time. Then, model (9) after adding the control mechanism is given as follows:

(14) $$\begin{array}{c} \left\{\begin{array}{c} \frac{dS_{k}\left(t\right)}{dt}=B-\alpha kS_{k}\left(t\right)\frac{1}{\langle k\rangle }\sum_{k=1}^{n}kp\left(k\right)I_{k}\left(t\right)-\mu S_{k}\left(t\right)+\varphi U_{k}\left(t\right)L_{k}\left(t\right),\\ \frac{dH_{k}\left(t\right)}{dt}=\theta_{2}\alpha kS_{k}\left(t\right)\frac{1}{\langle k\rangle }\sum_{k=1}^{n}kp\left(k\right)I_{k}\left(t\right)-\left(\beta +\mu \right)H_{k}\left(t\right),\\ \frac{dI_{k}\left(t\right)}{dt}=\theta_{1}\alpha kS_{k}\left(t\right)\frac{1}{\langle k\rangle }\sum_{k=1}^{n}kp\left(k\right)I_{k}\left(t\right)+m\beta H_{k}\left(t\right)-\left(\gamma +\mu \right)I_{k}\left(t\right)+\varphi \left(1-U_{k}\left(t\right)\right)L_{k}\left(t\right),\\ \frac{dL_{k}\left(t\right)}{dt}=\gamma I_{k}\left(t\right)-\left(\varphi +\eta +\mu \right)L_{k}\left(t\right). \end{array}\right. \end{array}$$

Defining the objective function as follows:

(15) $$\begin{array}{c} J\left(U\left(t\right)\right)=\int_{0}^{T}\sum_{k=1}^{n}\left(B_{1}I_{k}\left(t\right)+B_{2}U_{k}^{2}\left(t\right)\right)dt, \end{array}$$

where $B_{1}$ is the positive balancing coefficient of infected individual density $I_{k}\left(t\right)$, and $B_{2}$ is the positive weight coefficient of control costs. In addition, $U\left(t\right)=(U_{1}\left(t\right),U_{2}\left(t\right),\cdots ,U_{n}\left(t\right)).$ From [43], for the objective function (15) subject to model (14), there exists an optimal control $U^{*}\left(t\right)=\left(U_{1}^{*}\left(t\right),U_{2}^{*}\left(t\right),\cdots ,U_{n}^{*}\left(t\right)\right)\in \Delta$ such that $J\left(U^{*}\left(t\right)\right)=\min J\left(U\left(t\right)\right)$. Therefore, this optimal control problem can be solved with the help of Pontryagin’s Maximum Principle. Define the Lagrangian as:

$$L\left(I_{k}\left(t\right),U\left(t\right)\right)=\sum_{k=1}^{n}\left(B_{1}I_{k}\left(t\right)+B_{2}U_{k}^{2}\left(t\right)\right).$$

In addition, Hamiltonian function can be defined as:

$$\begin{array}{cc} \; & Q(S_{k}\left(t\right),H_{k}\left(t\right),I_{k}\left(t\right),L_{k}\left(t\right),U\left(t\right),\lambda_{j}\left(t\right))\\ \; & =\sum_{k=1}^{n}Q_{k}\left(S_{k}\left(t\right),H_{k}\left(t\right),I_{k}\left(t\right),L_{k}\left(t\right),U\left(t\right),\lambda_{jk}\left(t\right)\right)\\ \; & =L\left(I_{k}\left(t\right),U\left(t\right)\right)+\sum_{k=1}^{n}\left(\lambda_{1k}\left(t\right)\frac{dS_{k}\left(t\right)}{dt}+\lambda_{2k}\left(t\right)\frac{dH_{k}\left(t\right)}{dt}+\lambda_{3k}\left(t\right)\frac{dI_{k}\left(t\right)}{dt}+\lambda_{4k}\left(t\right)\frac{dL_{k}\left(t\right)}{dt}\right), \end{array}$$

where j=1,2,3,4. $\lambda_{jk}\left(t\right)$ is a Lagrange multiplier with k=1,2,⋯,n. After the above analysis, one can draw the following conclusions.

**Theorem** **3.** *The optimal control problems* (14) *and* (15) admit an optimal solution $\left({\hat{S}}_{k}\left(t\right),{\hat{H}}_{k}\left(t\right),{\hat{I}}_{k}\left(t\right),{\hat{L}}_{k}\left(t\right)\right)$ accompanied by the optimal control $U_{k}^{*}\left(t\right)$ on (0,T) for k=1,2,⋯,n.

The optimal media report control strategy $U_{k}^{*}\left(t\right)$ is computed by:

$$U_{k}^{*}\left(t\right)=\max\left\{\min\left\{1,\frac{\left(\lambda_{3k}\left(t\right)-\lambda_{1k}\left(t\right)\right)\varphi L_{k}\left(t\right)}{2B_{2}}\right\},0\right\},\;\;k=1,2,\cdots ,n.$$

*where the adjoint function $\lambda_{jk}\left(t\right)\left(j=1,2,3,4,\;\;k=1,2,\cdots ,n.\right)$ satisfying:*

$$\begin{array}{c} \left\{\begin{array}{cc} \frac{d\lambda_{1k}\left(t\right)}{dt}= & \lambda_{1k}\left(t\right)\alpha k\sum_{k=1}^{n}kp\left(k\right)\frac{1}{\langle k\rangle }{\hat{I}}_{k}\left(t\right)+\mu \lambda_{1k}\left(t\right)-\lambda_{2k}\left(t\right)\theta_{2}\alpha k\sum_{k=1}^{n}kp\left(k\right)\frac{1}{\langle k\rangle }{\hat{I}}_{k}\left(t\right)\\ \; & -\lambda_{3k}\left(t\right)\theta_{1}\alpha k\sum_{k=1}^{n}kp\left(k\right)\frac{1}{\langle k\rangle }{\hat{I}}_{k}\left(t\right),\\ \frac{d\lambda_{2k}\left(t\right)}{dt}= & \left(\beta +\mu \right)\lambda_{2k}\left(t\right)-m\beta \lambda_{3k}\left(t\right),\\ \frac{d\lambda_{3k}\left(t\right)}{dt}= & -B_{1}+\lambda_{1k}\left(t\right)\alpha k_{k}\left(t\right)\frac{kp(k)}{\langle k\rangle }{\hat{S}}_{k}\left(t\right)-\lambda_{2k}\left(t\right)\theta_{2}\alpha k\frac{kp(k)}{\langle k\rangle }{\hat{S}}_{k}\left(t\right))\\ \; & -\lambda_{3k}\left(t\right)\theta_{1}\alpha k\frac{kp(k)}{\langle k\rangle }{\hat{S}}_{k}\left(t\right)+\left(\mu +\gamma \right)\lambda_{3k}\left(t\right)-\gamma \lambda_{4k}\left(t\right),\\ \frac{d\lambda_{4k}\left(t\right)}{dt}= & \left(\lambda_{3k}\left(t\right)-\lambda_{1k}\left(t\right)\right)\varphi U_{k}^{*}\left(t\right)-\varphi \lambda_{3k}\left(t\right)+\lambda_{4k}\left(t\right)\left(\varphi +\eta +\mu \right). \end{array}\right. \end{array}$$

*The transversality conditions are $\lambda_{jk}\left(T\right)=0,\;\;j=1,2,3,4$.*

**Proof** **of Theorem 3.**  By the optimal conditions, one can obtain

$$\begin{array}{cc} \; & \frac{\partial Q}{\partial U_{k}}{|}_{S_{k}\left(t\right)=S_{k}^{*},\;H_{k}\left(t\right)=H_{k}^{*},\;I_{k}\left(t\right)=I_{k}^{*},\;L_{k}\left(t\right)=L_{k}^{*}}\\ \; & =2B_{2}U_{k}^{*}\left(t\right)+\lambda_{1k}\left(t\right)\varphi L_{k}\left(t\right)-\lambda_{3k}\left(t\right)\varphi L_{k}\left(t\right)=0. \end{array}$$

Thus, $U_{k}^{*}\left(t\right)=\frac{\left(\lambda_{3k}\left(t\right)-\lambda_{1k}\left(t\right)\right)\varphi L_{k}\left(t\right)}{2B_{2}}.$

Considering the properties of Hamiltonian functions and the range of the control variable, one obtains:

(16) $$\begin{array}{c} U_{k}^{*}\left(t\right)=\left\{\begin{array}{cc} 0, & \mathrm{if}\;\frac{\left(\lambda_{3k}\left(t\right)-\lambda_{1k}\left(t\right)\right)\varphi L_{k}\left(t\right)}{2B_{2}}\leq 0\\ \frac{\left(\lambda_{3k}\left(t\right)-\lambda_{1k}\left(t\right)\right)\varphi L_{k}\left(t\right)}{2B_{2}}, & \mathrm{if}\;0\leq \frac{\left(\lambda_{3k}\left(t\right)-\lambda_{1k}\left(t\right)\right)\varphi L_{k}\left(t\right)}{2B_{2}}\leq 1\\ 1, & \mathrm{if}\;\frac{\left(\lambda_{3k}\left(t\right)-\lambda_{1k}\left(t\right)\right)\varphi L_{k}\left(t\right)}{2B_{2}}>1. \end{array}\right. \end{array}$$

Therefore, the optimal control is obtained as follows:

$$U_{k}^{*}\left(t\right)=\max\left\{\min\left\{1,\frac{\left(\lambda_{3k}\left(t\right)-\lambda_{1k}\left(t\right)\right)\varphi L_{k}\left(t\right)}{2B_{2}}\right\},0\right\},\;\;k=1,2,\cdots ,n.$$

□

## 5. Numerical Simulations

In this section, several numerical examples are given to test the correctness of the above theoretical results. Our studies are performed in a BA scale-free network with a power law degree distribution: $p\left(k\right)=\frac{2l(l+1)}{k(k+1)(k+2)}\propto 2l^{2}k^{-3}$, where *l* is the minimum degree of the addressed network. Select the maximum degree of the nodes in the networks as 100. The calculation shows that $\langle k\rangle =\sum_{k=1}^{n}kp\left(k\right)=3.27.$

In order to study the role of basic reproduction number $R_{0}$ on rumor propagation, we chose two sets of parameter values to explore the process of rumor propagation when the basic reproduction number $R_{0}<1$ and $R_{0}>1$.

**Figure 2:**
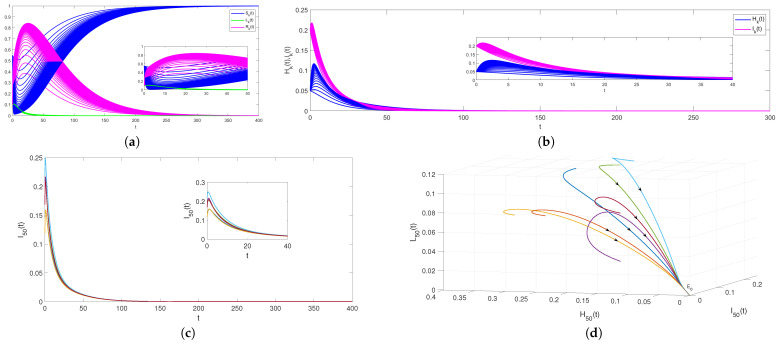
(**a**–**d**) The stability of $E_{0}$ for model (9) with $R_{0}<1$.

Firstly, take $n=100,B=0.02,\alpha =0.2,\beta =0.1,\gamma =0.16,\mu =0.02,\theta_{1}=0.15,\theta_{2}=0.2,\eta =0.2,\varphi =0.1,m=0.02$. After simple calculation, we can get $R_{0}=0.7484<1$. Based on Theorem 1, the rumor-free equilibrium $E_{0}$ is globally asymptotically stable as depicted in Figure 2. Figure 2a,b shows the evolution of $\left(S_{k}\left(t\right),H_{k}\left(t\right),I_{k}\left(t\right),L_{k}\left(t\right),R_{k}\left(t\right)\right)$ with degree taking from 1 to 50. Thus, we can draw a conclusion that even with different values for *k*, rumors will die out eventually and the density of infected individuals will converge to zero when $R_{0}<1$. Figure 2c,d is drawn by fixing the degree k=50 and keeping other parameter values the same as in Figure 2a,b. The evolution of $\left(H_{50}\left(t\right),I_{50}\left(t\right),L_{50}\left(t\right)\right)$ under different initial values shows that the rumor will die out eventually. It should be noted that all state trajectories converge to rumor-free equilibrium $E_{0}$, so rumor-free equilibrium $E_{0}$ is globally asymptotically stable.

**Figure 3:**
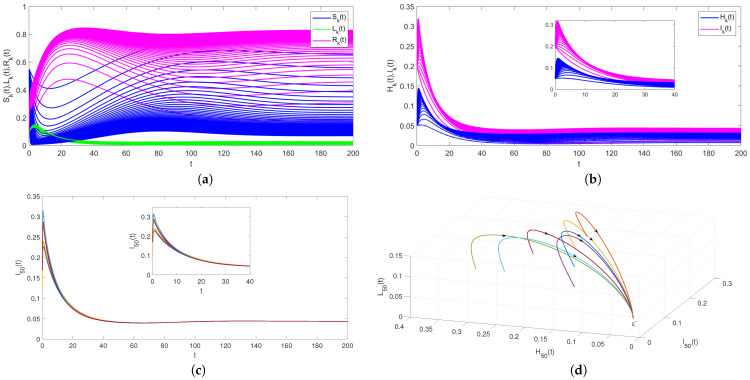
(**a**–**d**) The stability of $E^{*}$ for model (9) with $R_{0}>1$.

Secondly, take $n=100,B=0.02,\alpha =0.35,\beta =0.1,\gamma =0.2,\mu =0.02,\theta_{1}=0.3,\theta_{2}=0.2,\eta =0.2,\varphi =0.1,m=0.4$, which results in $R_{0}=2.5852>1$. Based on Theorem 2, the rumor-spreading equilibrium $E^{*}$ is globally asymptotically stable as depicted in Figure 3. Figure 3a,b shows the evolution of $\left(S_{k}\left(t\right),H_{k}\left(t\right),I_{k}\left(t\right),L_{k}\left(t\right),R_{k}\left(t\right)\right)$ with degree taking from 1 to 50. These two pictures show that with regard to different values for *k*, rumors will continue to prevail and the density of all state individuals will converge to $E^{*}$ when $R_{0}>1$. Furthermore, with the increase of degree *k*, the peak density and the final scale of rumor infected individuals also increase. In real life, everyone has a different number of friends. Those with good social skills have a greater impact on the spread of rumors, which is more realistic. Additionally, Figure 3c,d is drawn by fixing the degree k=50 and keeping other parameter values the same as Figure 3a,b. The evolution of $\left(H_{50}\left(t\right),I_{50}\left(t\right),L_{50}\left(t\right)\right)$ under different initial values shows that the rumor will continue to prevail. Note that all state trajectories converge to rumor-spreading equilibrium $E^{*}$, so rumor-spreading equilibrium $E^{*}$ is globally asymptotically stable.

**Figure 4:**
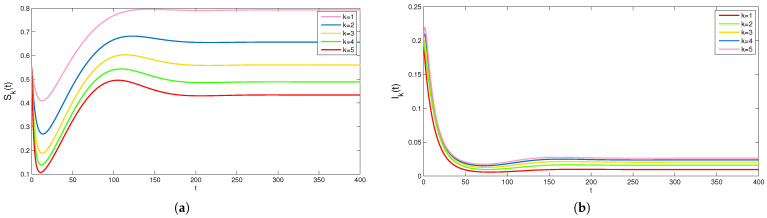
The densities of (**a**) $S_{k}\left(t\right)$ and (**b**) $I_{k}\left(t\right)$ with different values of *k*.

In the following, the effect of degree *k* on rumor propagation is studied. Choose $B=0.02,\alpha =0.35,\beta =0.1,\gamma =0.2,\mu =0.02,\theta_{1}=0.3,\theta_{2}=0.2,\eta =0.2,\varphi =0.1,m=0.4$. Fix the set of parameter values of $R_{0}>1$ except degree *k* changes from 1 to 5. In this case, the rumor will continue spreading. The plots in Figure 4 show that the peak density of $S_{k}\left(t\right)$ decreases with increasing *k* and the peak density of $I_{k}\left(t\right)$ increases with increasing *k*. This means the degree of the network will affect the maximum scale of rumor spread. The greater the degree of the network, the larger the scale of rumor spreading. Generally speaking, degree *k* is conducive to the rumor propagation.

**Figure 5:**
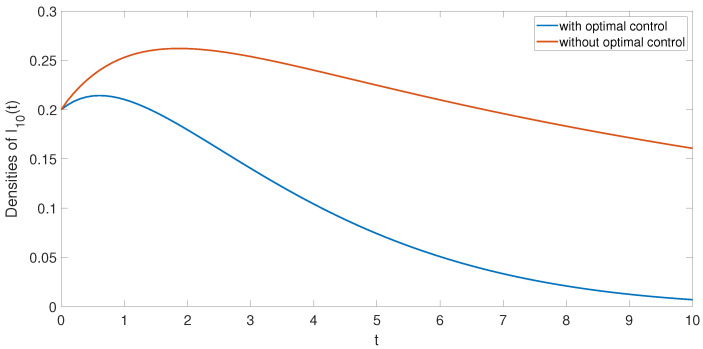
$I_{10}\left(t\right)$ with optimal control (blue) and without optimal control (red).

Considering the controlled system (14), the parameters are shown as following. Choose n=100, $B=0.02,\;\alpha =0.35,\;\beta =0.1,\;\gamma =0.2,\;\mu =0.02,\;\theta_{1}=0.3,\;\theta_{2}=0.2,\;\eta =0.2,\;\varphi =0.18,$ m=0.4, $B_{1}=5,\;B_{2}=0.2$, which leads to rumor propagation. In order to study the influence of optimal control on system (14), we simulate the trajectories of system (14) with and without optimal control, as shown in Figure 5. Through observation, we find that the density of rumor infected individuals under optimal control is lower than that of rumor infected individuals without optimal control. In other words, the density of infected individuals decreases under optimal control. Therefore, we can draw the conclusion that the optimal control strategy proposed in this paper can effectively suppress the spread of rumors.

**Figure 6:**
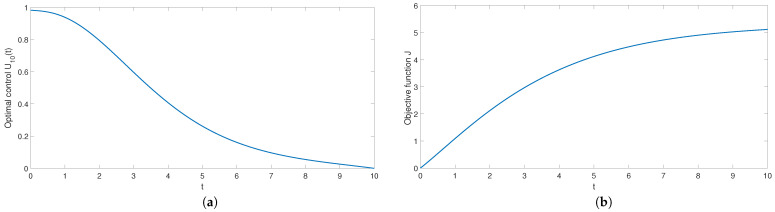
The path of optimal control (**a**) and control costs (**b**).

The trajectories of optimal control and control cost over time are shown in Figure 6a,b, respectively. It can be seen in Figure 6a that the control intensity is larger at the initial time, and the control intensity gradually decreases to 0. This means that it is easier to control rumor propagation later after high control investment in the early stage. In other words, in the process of rumor propagation, the control intensity will gradually decrease. It can be seen in Figure 6b that control consumption J(t) reaches the maximum value at t=10, which is in line with the actual situation. In actuality, total control consumption will increase with the increase of control time.

## 6. Model Application

In this section, a practical example is used to verify the validity of the theoretical results.

This paper uses the actual data supplied in Hu and Zhao [44], which presents a rumor about haze. Relevant data about the number of people spreading rumors over time is shown in Table 1. In particular, it is assumed that the rumor is spread without rumor recurrence.

**Figure 7:**
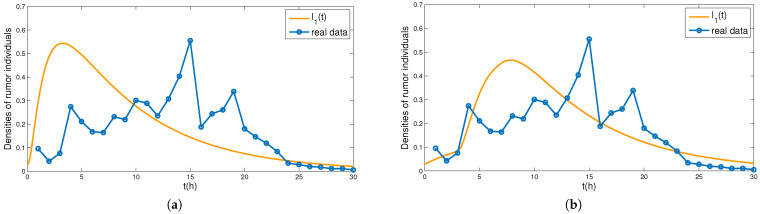
(**a**,**b**) Number of individuals spreading the rumor.

In Figure 7, we fit the established model (4) with the real rumor propagation example. In Figure 7a, we take $\alpha =0.95,\;\beta =0.1,\;\gamma =0.16,\mu =0.002,\;\theta_{1}=0.85,\;\theta_{2}=0.02,$ η=0.7, φ=0.1, m=0.02. It is found that the data fitting effect is better from the 20th hour of rumor spreading. In the early stage of rumor propagation, the established models obviously peak earlier than the rumor in the practical example. The reason for this may be time delay in rumor propagation, we simulate the case where time delay is equal to three in Figure 7b. Time delay can not only prolong the outbreak time of rumors, but can also reduce the peak of rumors.

**Remark** **7.** *The numerical fitting results of real cases indicate that time delay has a great impact on the spread of rumors. Time delay not only can prolong the outbreak time of rumors, but also reduce the peak density of I(t). Therefore, it is necessary to take time delay into account in future research.*

**Figure 8:**
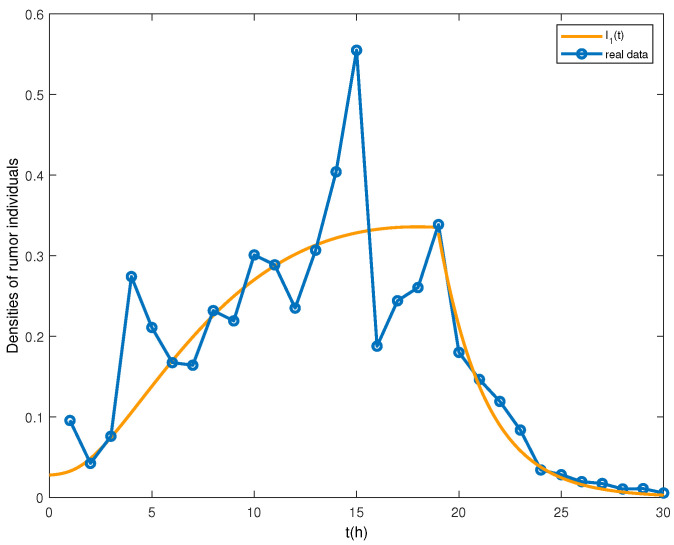
Number of individuals spreading rumors.

From Table 1, it is found that when *T* = 20 h, the rumor quickly vanished with the intervention of the media. Therefore, we apply the control proposed in system (14) at *T* = 20 h to reveal the evolution of rumor individual density. The densities of rumor-spreading individuals can be seen in Figure 8. Obviously, the path with control is highly consistent with the actual data. Therefore, the control strategy is effective.

**Remark** **8.** *This paper studies the dynamics of rumor propagation in heterogeneous networks. In fact, there are many factors to be taken into consideration, such as reaction–diffusion in space [45], time delays [46] and so on. In addition, it is helpful for us to consider several novel rumor propagation mechanisms to understand rumor propagation. Recently, Zhu et al. discussed individuals’ dynamic friendship in [47], and Liu et al. considered competitive information dissemination in [48]. These works have broadened our research perspective. There is an expectation to implement more laws to control rumor propagation so as to better control rumors. Therefore, it is necessary to take the above factors into consideration in future work.*

## 7. Conclusions

In this paper, a novel rumor spreading model with consideration of recurrence, individual behavior and an optimal media coverage control strategy is proposed in heterogeneous networks (e.g., BA scale-free network). The dynamics of the model are explored with the help of the mean field equation. Some useful conclusions are drawn in order to ensure globally asymptotically stable rumor-free equilibrium and rumor-spreading equilibrium in heterogeneous networks with Lyapunov method and LaSalle’s Invariance Principle. In addition, optimal control is acquired by taking advantage of Pontryagin’s Maximum Principle. The results are checked through a number of experiments. Moreover, based on practice, a model application is presented to explain the rationality of the established model and the proposed optimal control. Furthermore, in the future, we will research the dynamics of the SHILR model with time delays and age structure, which is more complicated but more realistic.

## Figures and Tables

**Table 1:** Number of individuals spreading the rumor.

Time	1 h	2 h	3 h	4 h	5 h	6 h	7 h	8 h	9 h	10 h
Number	210	93	167	603	464	368	361	511	480	662
Time	11 h	12 h	13 h	14 h	15 h	16 h	17 h	18 h	19 h	20 h
Number	635	517	675	889	1221	413	557	573	745	396
Time	21 h	22 h	23 h	24 h	25 h	26 h	27 h	28 h	29 h	30 h
Number	322	262	184	75	62	43	38	23	24	12

## Data Availability

Not applicable.
